# *Centaurea pumilio* (Asteraceae): Conservation Status, Threats and Population Size of a Critically Endangered Species in Italy

**DOI:** 10.3390/plants14071074

**Published:** 2025-04-01

**Authors:** Alessio Turco, Robert Philipp Wagensommer, Pietro Medagli, Saverio D’Emerico, Fabio Ippolito, Giuseppe Scordella, Antonella Albano

**Affiliations:** 1 Faculty of Education, Free University of Bozen-Bolzano, 39042 Bressanone, BZ, Italy; alessio.turco@unibz.it; 2 Department of Biological and Environmental Sciences and Technologies, University of the Salento, 73100 Lecce, LE, Italy; pietro.medagli@unisalento.it (P.M.); fabio.ippolito@unisalento.it (F.I.); antonella.albano@unisalento.it (A.A.); 3 “Aldo Moro” University of Bari, 70125 Bari, BA, Italy; sdeme@yahoo.it; 4 “Litorale di Ugento” Regional Natural Park, Piazza Colosso 1, 73059 Ugento, LE, Italy; giuseppe.scordella@comune.ugento.le.it

**Keywords:** conservation, drone, GIS, population size, red list, threats

## Abstract

This paper presents a comprehensive study of the size and conservation status of the only Italian population of *Centaurea pumilio* (Asteraceae) and the threats to its survival. The population is located on a short stretch of sandy shoreline along the Ionian coast of Puglia, near Torre S. Giovanni (Ugento, Lecce). It was estimated in the 1990s to number about 500 plants, but in recent years a significant reduction, bringing the population to fewer than 100 individuals, has been observed. This study involved a census of the individuals (differentiating young plants from adult and reproductive ones) conducted with a precision GPS tool, phytosociological analysis and high-definition orthophoto image acquisition using a drone. Concerning the latter, to evaluate anthropic pressure from tourism, data were acquired in spring 2023 and autumn 2024 and compared using GIS geoprocessing, showing a significant increase in the area occupied by footpaths. GIS analysis also revealed that the survival of *C. pumilio* is strongly linked to the intensity of the walking routes, which have fragmented the population into small and isolated clusters. On the basis of all the collected data, the current conservation status of the species in Italy was assessed as critically endangered. Finally, our study provides a series of suggestions to improve the conservation status of the species and strategies to reduce the risk of extinction in Italy.

## 1. Introduction

*Centaurea* L. is one of the richest and taxonomically intricate genera of the family Asteraceae, comprising between 400 and 700 species, distributed predominantly in the Old World, mainly north of the equator and in the eastern hemisphere [1,2,3]. Eastern Anatolia and the Transcaucasus are the primary centres of origin and diversity, while the Mediterranean area and the Balkan Peninsula are secondary [2]. The Middle East and its surrounding regions are particularly rich in *Centaurea* species, with Turkey hosting 166 species [3,4,5]. The species of the genus are therophytes, hemicryptophytes, chamaephytes or more rarely nanophanerophytes, with alternate pinnatifid to pinnatipartite leaves. The flower-head bracts are multi-seriate and imbricate, having a membranous or slender appendage, often hard, spiny or bristly at the apex. The flowers are pink, blackish-purple, blue, yellow or whitish [5].

In the native vascular flora of Italy, the genus *Centaurea* is represented by 119 confirmed taxa (species and subspecies), of which 78 are endemic, plus two doubtfully occurring taxa and one species no longer recorded [6]. Puglia hosts 23 *Centaurea* taxa, nine of which are endemic to Italy, with five of these endemic to Puglia. In addition, three *Centaurea* taxa are doubtful in Puglia, while one taxon is no longer recorded [6]. Most of the *Centaurea* species endemic to Puglia occur in the Salento peninsula [7,8].

**Figure 1:**
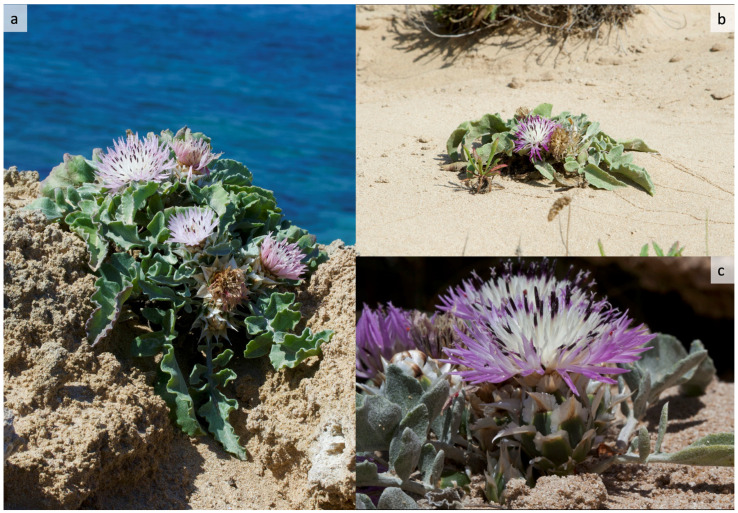
*Centaurea pumilio* L. at its only Italian site (Torre San Giovanni, Ugento–Lecce, Puglia). (**a**,**b**) Habitat and habitus of the species; (**c**) Close-up of the inflorescence, showing the involucral bracts.

*Centaurea pumilio* L. (≡ *Aegialophila pumilio* (L.) Boiss.) (Figure 1) is a very rare species in Italy, occurring only in Puglia at Torre San Giovanni (Ugento, Lecce), along the Ionian coast of Salento, where it was first reported in 1996 [9,10]. Although POWO [11] considers *C. pumilio* to be a synonym of *Crocodilium pumilio* (L.) N. Garcia & Susanna, we prefer to include it in the genus *Centaurea*, in accordance with the recent checklist of vascular flora native to Italy [6]. It is a scapose hemicryptophyte characterised by a reduced stem, 4–20 cm tall, flowering in April and May on maritime sands [9,10,12]. It is a psammophilous plant, with an Eastern Mediterranean distribution, occurring on sandy coasts from Northern Africa (Libya and Egypt) to Israel and Syria and in Europe in Greece (Crete, the Ionian islands and the Peloponnese) and Italy (Puglia) [13,14]. The Italian site therefore represents the north-western limit of the species’ geographical distribution. Interestingly, it is not the only plant taxon of phytogeographic interest for which Puglia represents the western limit of its Eastern Mediterranean distribution [15,16,17]. It is nevertheless rare and often endangered [13]. Along the coast of Libya, *C. pumilio* is found in association with *Thinopyrum junceum* (L.) Á. Löve [18]. In Israel, it is included in the national red list [19]. In Crete, it is associated with *Thinopyrum junceum* and *Medicago marina* L. [13]. In addition to habitat destruction, *C. pumilio* is endangered by harvesting, as in Egypt, where the roots are collected for traditional medicinal use [20].

In Italy, the environment where *C. pumilio* grows is very similar to that of the southeast Mediterranean basin, and the Puglia population might thus represent a relict site of the species’ original distribution [21]. In fact, although POWO [11] considers *C. pumilio* to be alien to Italy, it is usually considered native to Italy [6,22,23]. The conservation status of the species in Italy was assessed as critically endangered (CR) [22,23], based on the single site of occurrence having an area of less than 10 km^2^, with the number of mature individuals estimated at fewer than 50 and in continuous decline. Other plant species with a very limited distribution and a low number of individuals have been assessed as endangered or critically endangered, both in Italy [24,25] and in other countries [26,27].

The aim of this study was to obtain updated information on the size and conservation status of the Italian population, together with any threats to it, using a multidisciplinary approach. This entailed the following:

(a) Characterisation of the vegetational features of the phytocoenosis in which the species lives;
(b) Updating its Italian distribution, analysing the changes in population density since its discovery;
(c) Updating its conservation status in Italy, assessed in accordance with IUCN categories and criteria;
(d) Landscape analysis to describe and evaluate the impact of anthropic pressure during the summer months, based on high-resolution orthophotos taken before (spring 2023) and after the summer season (autumn 2024).

Finally, our study provides a series of suggestions to improve the plant’s conservation status and strategies to reduce the risk of extinction in Italy.

## 2. Materials and Methods

### 2.1. Study Area

**Figure 2:**
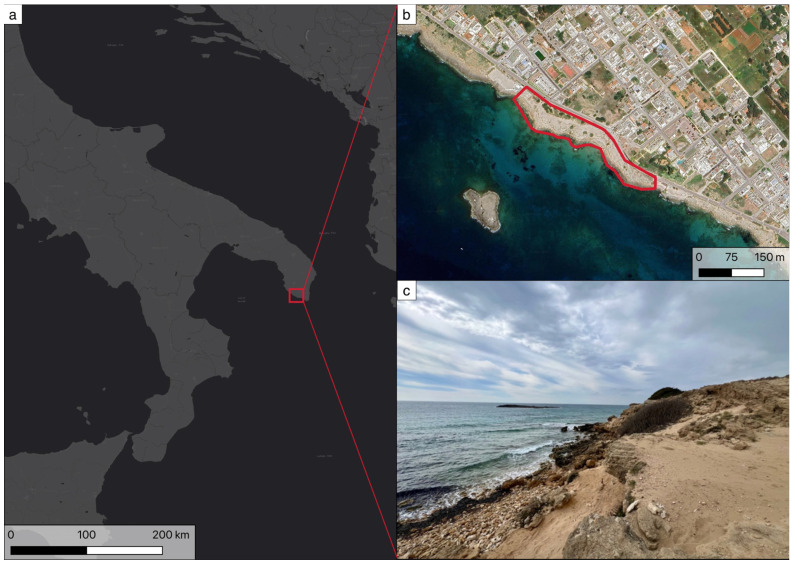
Study area: (**a**) The Ionian coast of Puglia; (**b**) Location of the small area in which *Centaurea pumilio* lives in Italy; (**c**) Photo of the study area in May 2023.

The only site of occurrence of *C. pumilio* in Italy is located along the Ionian coast of Puglia, near Torre S. Giovanni (Ugento, Lecce), within the boundaries of the “Litorale di Ugento” Regional Natural Park, an area rich in plant species and endemism, including orchids [28]. The species grows on a sandy substrate, deriving from the degradation of compact sands of sedimentary origin, which rest on Holocene calcarenites (Figure 2). In the past, this area was also affected by an archaeological excavation, which brought to light a settlement from the Middle Bronze Age [29].

From a vegetational point of view, the area, which is subject to high anthropic pressure due to use by beachgoers, is partially covered by a sclerophyllous scrub community belonging to the order *Pistacio lentisci-Rhamnetalia alaterni* Rivas-Martínez 1975, corresponding to habitat 2260 (*Cisto-Lavanduletalia* dune sclerophyllous scrubs) of the 92/43/EEC Directive. The rest of the sand dunes have very fragmented, sparse and discontinuous vegetation belonging to the alliance *Agropyrion juncei* (Tüxen in Br.-Bl. & Tüxen 1952) Géhu, Rivas-Martínez & Tüxen 1972 in Géhu, Costa, Scoppola, Biondi, Marchiori, Peris, Franck, Caniglia & Veri 1984, corresponding to habitat 2110 (embryonic shifting dunes) of the 92/43/EEC Directive.

### 2.2. Vegetation Investigation

To clarify the syntaxonomic position of the plant community in which *C. pumilio* lives, three phytosociological relevés were carried out in May 2023.

The phytosociological survey was conducted in accordance with the methods of the Zurich–Montpellier school [30]. All vascular plants were recorded using the standard seven-grade Braun–Blanquet scale for estimating cover and abundance: r = coverage <1% with very few individuals; + = coverage <1% with a moderate number of individuals; 1 = coverage between 1% and 5%; 2 = coverage between 6% and 25%; 3 = coverage between 26% and 50%; 4 = coverage between 51% and 75%; 5 = coverage between 76% and 100%.

The nomenclature of the species follows the updated checklist of vascular flora native to Italy [6].

### 2.3. Acquisition of Current and Previous Distribution Data

In May 2023 a census of the population of *C. pumilio* was carried out. Using the Differential GPS Geomax Zenith35 Pro GSM-UHF-TAG (GeoMax AG manufacturers, Widnau, Switzerland), the position of each plant was recorded, distinguishing young plants that had not yet flowered from mature and flowering plants.

To evaluate variations in population density and location since its discovery in 1996 [9,10], distribution data for *C. pumilio* were taken from a previous unpublished census that we performed in 2007, which also separated young plants from mature ones. The 2023 survey of *C. pumilio* individuals used a georeferenced grid constructed in a GIS environment, in which each grid square represented a unit of land measuring 1.47 m × 1.47 m, thereby replicating the methodology of the 2007 census and enabling a comparison of the data. The grid was obtained by taking the Gauss–Boaga grid at a scale of 1:10,000 as a reference and dividing it into increasingly smaller regular square portions until reaching the size of the land unit.

The data collected in 2007 were converted to the WGS84 reference system and used in a GIS environment to compare them with the recent census of 2023.

The cartography was produced using QGis version 3.34.14-Prizren [31].

### 2.4. Conservation Status Assessment

The conservation status of the species in Italy was assessed in accordance with IUCN categories and criteria [32], adapted for sub-global assessments [33], according to which downlisting is necessary for taxa that experience significant immigration that is not expected to decrease and uplisting is necessary if the regional population is a sink and immigration is expected to decrease.

### 2.5. Orthophoto Acquisition

To obtain up-to-date information on the distribution of the only Italian population of *C. pumilio* and evaluate pressures and threats to its survival, high-definition orthophotos were taken. The process was based on procedures for photogrammetric surveys, integrating images obtained using the RPA (Remotely Piloted Aircraft) DJI Phantom 4 PRO quadcopter drone (DJI manufacturers, Shenzhen, China), the surface mapping of topographic support points and polygonal correlation. The acquired dataset was managed using both specific multi-view 3D reconstruction software Agisoft Metashape (Professional Edition), to generate orthophotos and Digital Elevation Models (DEMs), and a GIS (Geographic Information System) environment, for editing the raster data (images) and vector data (support points).

The used aircraft acquired highly overlapping nadiral photographs during specific flight plans. The first field survey was conducted in June 2023, with a series of aerial photogrammetric flights aimed at reconstructing the geometries of the area. To evaluate variations in the fragmentation of the spontaneous vegetation and to measure the weight of human pressure from beachgoers during the summer months, a second drone flight was performed in October 2024. The definition of the orthophotos (1.4 cm/pixel) was much higher than that of the images made available [34] by the Puglia Region (20 cm/pixel).

## 3. Results

### 3.1. Vegetation Description

The results of the phytosociological survey (Table 1) show a floristically very poor psammophilous community. The number of species varies from two to five per phytosociological relevé. The vegetation is quite patchy, with cover ranging from 40% to 60%. Apart from *C. pumilio*, the most common or abundant species are *Thinopyrum junceum* (L.) Á. Löve, *Lotus creticus* L., *Plantago macrorhiza* Poir. and *Polycarpon tetraphyllum* (L.) L.

### 3.2. Analysis of Previous and Current Distribution Data

The first census of the population was conducted after its discovery in 1996 [9,10], when it was estimated to number “about 500 individuals, some of which (150) were of considerable size, distributed over a surface area of almost 2000 m^2^, with young individuals accounting for 80% of the total”.

**Figure 3:**
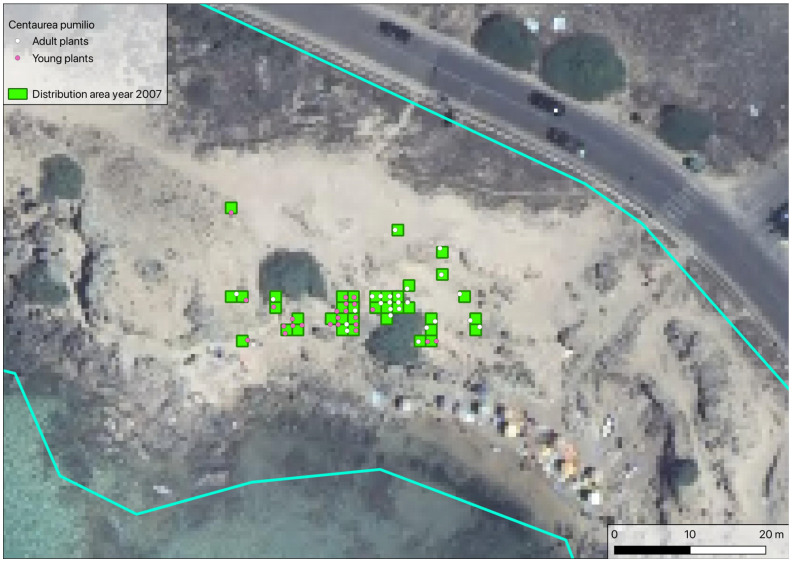
Location of the *Centaurea pumilio* population during the 2007 census. Blue lines: study area delimitation (as in Figure 2b).

During the second census of the population, performed in April–June 2007, a total of 285 plants were found, of which 65 were young and 220 adult. The surface area occupied by the population was estimated to be 79.95 m^2^, with young plants limited to an area of about 38.89 m^2^ (Figure 3).

**Figure 4:**
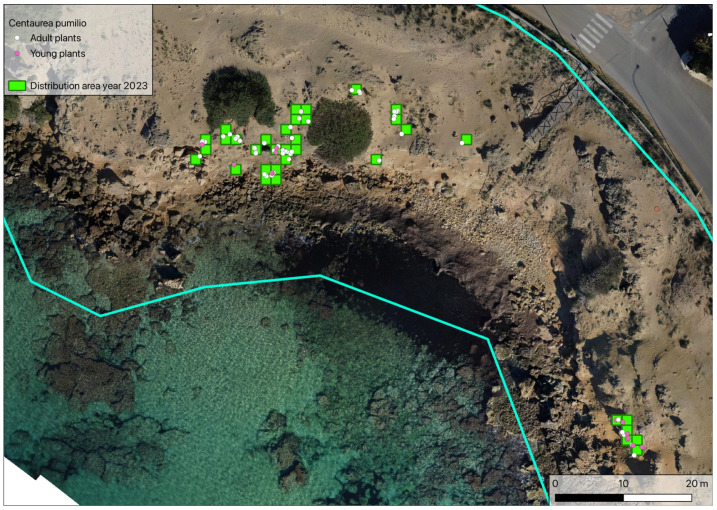
Location of the *Centaurea pumilio* population during the 2023 census. Blue lines: study area delimitation (as in Figure 2b).

During the third census of the population, performed in spring 2023, a total of 71 plants were found, of which 18 were young and 53 adult. During this census, a small new cluster of 13 individuals (5 adults and 8 young plants) was found on a small cliff with no other plant species present. The surface area occupied by the whole population was estimated to be 74.6 m^2^, with young plants limited to an area of about 23.77 m^2^ (Figure 4).

The population census carried out in autumn 2024 allowed us to identify only some of the plants already counted during spring 2023 (about 40%), most of which were the oldest and largest, which were re-growing after the summer drought period and waiting to flower the following spring. In contrast, few young plants were found.

Comparing previous with current distribution data, a significant decrease in population size and distribution area can be noted, in terms of both the number of individuals and the surface area occupied by the population (Table 2). With regard to the number of individuals, a decrease of 85.8% was observed, which was associated with a loss of surface area equal to 96.27%. Young plants show an even worse trend in terms of population reduction than adult ones, with a loss of 94.9–95.5%.

### 3.3. Conservation Status in Italy

The species is represented in Italy by a single, very small population, located in the Salento Peninsula (Puglia Region), occupying an area of less than 1 km^2^, with 53 mature individuals. The population is affected by tourism-related activities. A single location sensu IUCN [32] can be recognised. As a consequence, the species is confirmed as critically endangered (CR) in Italy on the basis of IUCN criteria B and C: CR B1ab(ii,iii,v)+2ab(ii,iii,v)+C2a(ii).

In accordance with the IUCN protocol for regional assessments [33], no downlisting was applied, given that no exchange is possible with populations occurring in other countries.

### 3.4. Orthophoto Analysis

**Figure 5:**
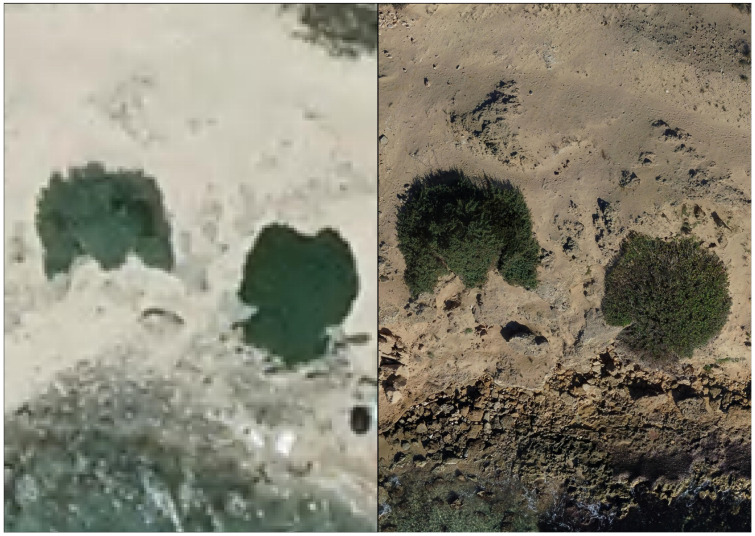
Orthophoto made available by the Puglia Region (2019 flight, **left**) and obtained by drone (autumn 2024 flight, **right**).

When compared to the orthophotos made available [34] by the Puglia Region (20 cm/pixel), the high definition of the orthophotos obtained with the drone flights of 2023 and 2024 (1.4 cm/pixel) allowed us to make more coherent and appropriate assessments of the conservation status of *C. pumilio* and of the threats affecting the species in Italy (Figure 5).

**Figure 6:**
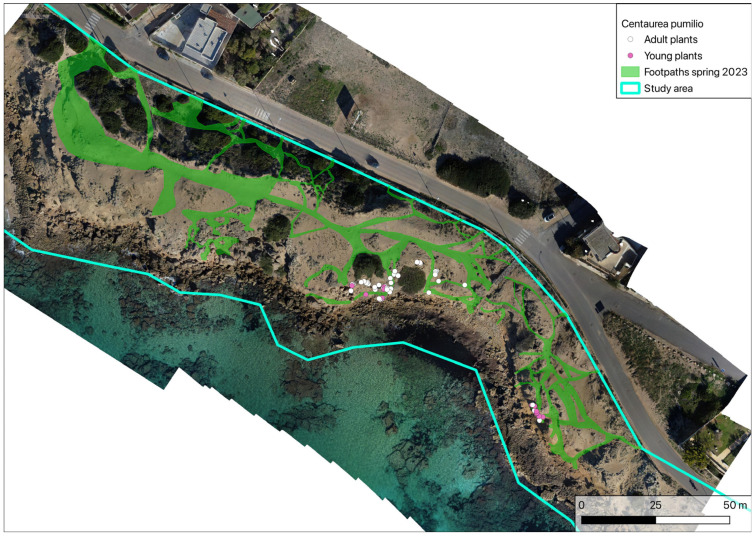
Analysis of footpaths in spring 2023.

Analysing the spring 2023 and autumn 2024 orthophotos, it was possible to trace and reconstruct the main anthropic pressures during the summer months based on the variation in the number, density and width of the footpaths on the sandy coastline. The surface area affected by footpaths in spring 2023 was estimated to be 2044 m^2^. Analysis of this area shows that all the vegetation, including its shrubby components, is characterised by high fragmentation (Figure 6).

**Figure 7:**
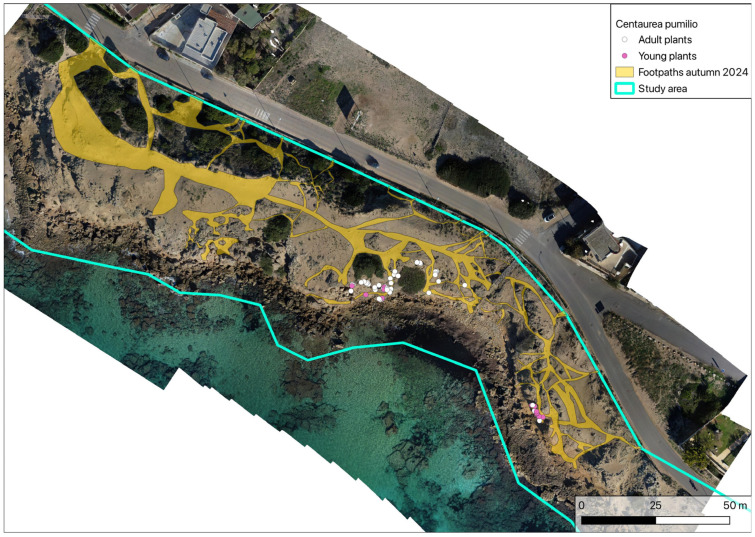
Analysis of footpaths in autumn 2024.

Analysis of the footpaths present in autumn 2024 (Figure 7) shows a clear worsening of the situation, since in a short time the area affected by the phenomenon rose to 2270 m^2^.

**Figure 8:**
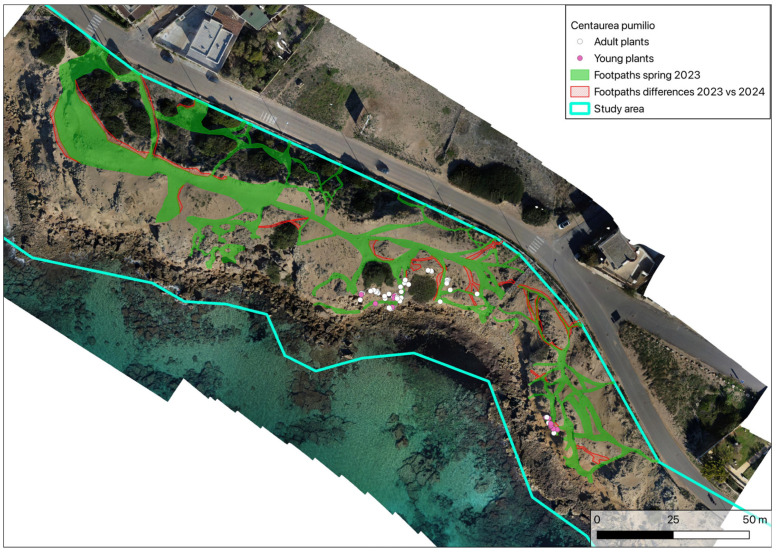
Difference between footpaths in spring 2023 vs. autumn 2024.

By means of GIS geoprocessing (based on the differences between polygons), a new map was drawn, making it possible to compare the footpaths of spring 2023 with those of autumn 2024. This showed that in this short period of time, there was an increase in the surface area taken up by footpaths of about 11% (Figure 8). This was mainly due to the expansion of existing footpaths but also the creation of new ones.

This analysis demonstrates that the entire study area is affected by strong pressure from trampling, mostly by beachgoers during the summer months.

## 4. Discussion

### 4.1. Vegetation Analysis

The presence of *Thinopyrum junceum* and *Lotus creticus* allows the plant community to be attributed to the suballiance *Agropyrenion farcti* Rivas-Martínez, Costa, Castroviejo & Valdés 1980, of the alliance *Agropyrion juncei* (Tüxen in Br.-Bl. & Tüxen 1952) Géhu, Rivas-Martínez & Tüxen 1972 in Géhu, Costa, Scoppola, Biondi, Marchiori, Peris, Franck, Caniglia & Veri 1984. This alliance is included in the order *Ammophiletalia australis* Br-Bl. 1933 and the class *Euphorbio paraliae-Ammophiletea australis* Géhu & Rivas-Martínez in Rivas-Martínez, Asensi, Díaz-Garretas, Molero, Valle, Cano, Costa & Díaz 2011 [35].

These psammophilous coenoses with few pioneer species correspond to habitat 2110 (embryonic shifting dunes), listed in Annex I of the 92/43/EEC Directive.

### 4.2. Conservation Status in Italy and Proposed Conservation Measures

The collected data allowed us to assess the species as critically endangered in Italy, confirming the previous assessments [22,23]. To avoid the extinction of *C. pumilio* in Italy, we propose the following in situ and ex situ conservation measures.

**Figure 9:**
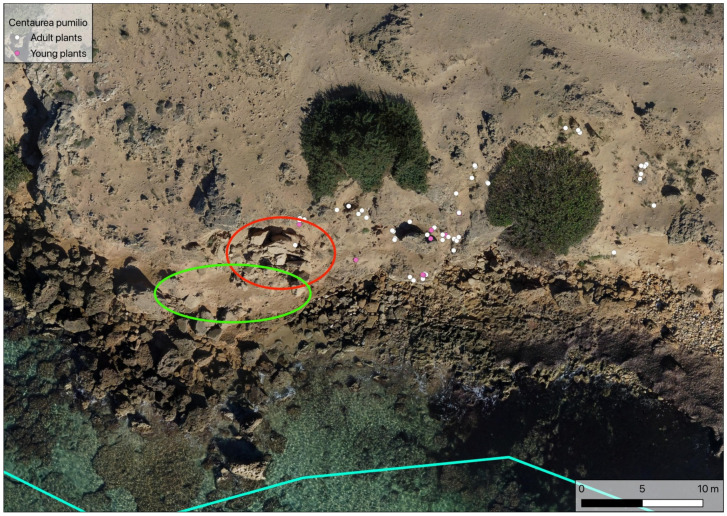
Identification of a portion of beach naturally closed to beachgoers (green circle), thanks to collapsed rocks (red circle). Blue line: study area delimitation (as in Figure 2b).

**Figure 10:**
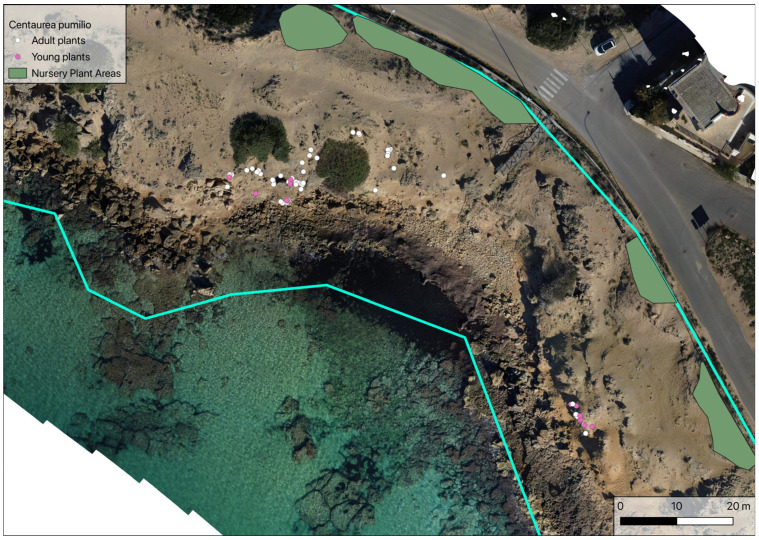
Identification of Nursery Plant Areas (green polygons). Blue lines: study area delimitation (as in Figure 2b).

In situ conservation measures:

- Identification of portions of the beach that are naturally inaccessible to beachgoers or can be closed off by positioning stone blocks; the sowing of *Centaurea pumilio* in these areas to allow the growth of seedlings under less anthropic pressure. A suitable area has already been identified (Figure 9, green circle), characterised by interruption of the coastline by fallen rocks (Figure 9, red circle);
- Protection of existing old plants at risk of being trampled, using plant cages to isolate small nuclei where it is not possible to exclude the passage of beachgoers. This must be evaluated very carefully and must be implemented at first on a small scale in order to observe the behaviour of beachgoers towards such deterrents and evaluate their effectiveness;
- Establishment of Nursery Plant Areas (NPAs), to promote the survival and dissemination of the species. In this case, macro-areas within which to sow *Centaurea pumilio* were identified. Areas not frequented by beachgoers, at a greater height above sea level than the existing population, were chosen (Figure 10). The latter condition will promote the rolling of the seeds towards the aforementioned areas with a view to increasing population density [12].

Ex situ conservation measures and translocation:

- Multiplication of the species in a Botanical Garden, aimed at strengthening the natural population or at introducing the species in another suitable area of the Ugento Regional Natural Park, to be carefully identified, the latter step being aimed at creating a new population in an area less affected by anthropic pressure.

### 4.3. Orthophoto Analysis and Footpaths Affecting Centaurea pumilio in Italy

**Figure 11:**
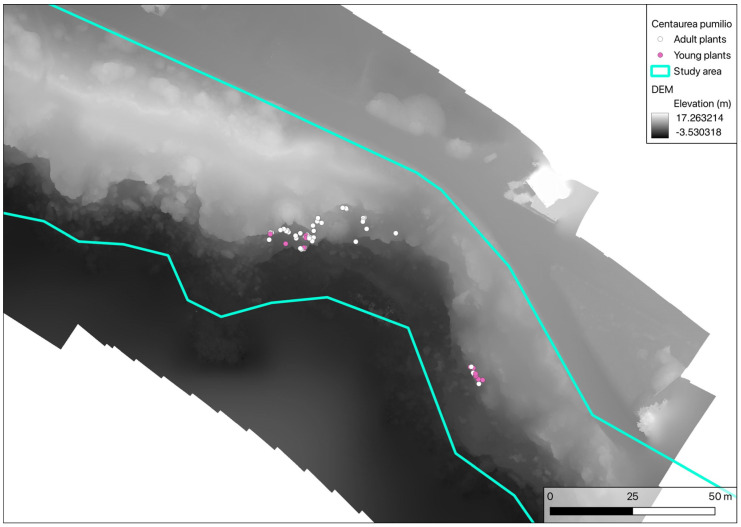
DEM (Digital Elevation Model) of the study area.

The reduction in the number of individuals and the occupied surface area (Table 2) is partially related to the geomorphology of the area. According to the Digital Elevation Model (DEM) obtained during orthophoto acquisition (Figure 11), the *C. pumilio* population is located at the base of a sand cliff, which effectively isolates and prevents the multiplication of the species. In fact, the pappuses are not able to fly, but can only roll downwards [12], in this case, into the sea.

An analysis of the footpaths (Figure 6 and Figure 7) shows that the entire coastline is affected by strong pressure from trampling by beachgoers during the summer months. All of the vegetation, both herbaceous and shrubby, is therefore characterised by high fragmentation, with consequent weakening of the entire dune cordon, an impoverishment of species diversity and a reduction in the capacity to resist further disturbance.

The greatest impact, progressively extinguishing the *C. pumilio* population in Italy, arises from the excessive human presence and consequently disturbance by trampling. In addition to damaging adult plants, this causes the destruction of young seedlings, which cannot find surfaces on which to develop undisturbed. The direct consequences of the increased area occupied by footpaths (Figure 8) are greater population fragmentation; isolation of adult plants (now confined to those parts of the beach that are inaccessible to beachgoers); prevention of germination; and burial of seedlings, mainly due to the excessive and continuous movement of sand caused by the passage of beachgoers.

**Figure 12:**
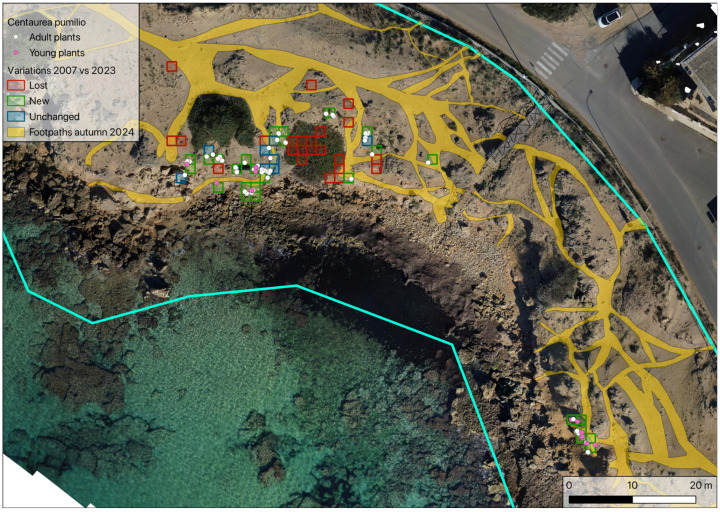
Comparison of footpaths in autumn 2024 and location of *Centaurea pumilio* plants in 2007 and 2023. Blue lines: study area delimitation (as in Figure 2b).

Indeed, comparing footpaths in 2024 with the location of *C. pumilio* plants in 2007 and 2023 (Figure 12), it is notable how the loss of plants is mainly due to beachgoers and only rarely due to the expansion of shrubs. Newly detected and preserved plants are located only along the edges of footpaths or in hard-to-reach places.

## 5. Conclusions

The tourism sector undoubtedly benefits the national economy, but many of its activities directly or indirectly contribute to the loss of biodiversity. The tourism sector and its associated activities have thus been recognised as having a negative impact on the environment and endangered species. Indeed, our study shows how intense human pressure in summer impacts negatively on the survival of Italy’s only *Centaurea pumilio* population.

The main tourism-related activities that lead to biodiversity loss can be classified as follows [36]: (i) habitat disruption due to the rapid and unplanned radical transformation of the landscape for tourism development (infrastructure and facilities); (ii) depletion of resources (e.g., water); (iii) littering and water pollution; (iv) sewage discharge from hotels, recreation facilities and other tourism-related structures; and (v) damage to coral reefs by careless tourists.

The case of *C. pumilio* in Italy certainly reflects the first point, i.e., habitat disruption, due to landscape transformation arising from trampling by beachgoers.

Our study describes the current conservation status of the only population of *C. pumilio* in Italy, demonstrating a significant decrease in population size, both in terms of the number of individuals and the presence of young plants, making it extremely difficult for the population to renew itself and/or expand.

The monitoring of the species in recent years has made it possible to understand the causes of its decline in Italy, allowing the development in the future of effective strategies for its conservation. Considering that the species is rare and often threatened in other sites outside Italy, our study could serve as a tool for the conservation of the species in other countries.

## Figures and Tables

**Table 1:** The *Centaurea pumilio* community in Italy.

Relevé Number	1	2	3
Date	31 May 2023	31 May 2023	31 May 2023
Latitude	39°53′46.35″	39°53′46.32″	39°53′46.82″
Longitude	18°05′57.83″	18°05′56.95″	18°05′56.88″
Elevation (m a.s.l.)	10	6	12
Area (m^2^)	1	2	1
Vegetation cover (%)	50	60	40
Average height of vegetation (cm)	35	30	20
Slope (°)	.	15	.
Aspect	.	S-SW	.
Number of species	2	5	4
			
*Centaurea pumilio* L.	3	2	2
			
**Characteristic species suballiance *Agropyrenion farcti***			
*Thinopyrum junceum* (L.) Á. Löve	3	3	+
*Lotus creticus* L.	.	2	.
			
**Other species**			
*Cutandia maritima* (L.) Benth. ex Barbey	.	+	.
*Plantago macrorhiza* Poir.	.	2	.
*Reichardia picroides* (L.) Roth	.	.	+
*Polycarpon tetraphyllum* (L.) L.	.	.	2

**Table 2:** Size and occupied area of the Italian *Centaurea pumilio* population over the years.

Year	Total Number of Plants	Number of Young Plants	Surface Area (m^2^)
1996	500	350–400	2000
2007	285	65	79.95
2023	71	18	74.6

## Data Availability

Data are contained in the article.
